# Single-particle mass analysis of intact ribosomes by mass photometry and Orbitrap-based charge detection mass spectrometry

**DOI:** 10.1016/j.isci.2021.103211

**Published:** 2021-10-02

**Authors:** Szu-Hsueh Lai, Sem Tamara, Albert J.R. Heck

**Affiliations:** 1 Biomolecular Mass Spectrometry and Proteomics, Bijvoet Center for Biomolecular Research and Utrecht Institute for Pharmaceutical Sciences, Utrecht University, 3584 CH Utrecht, The Netherlands; 2 Netherlands Proteomics Center, Padualaan 8, 3584 CH Utrecht, The Netherlands

**Keywords:** Physical chemistry, Biochemistry, Structural biology

## Abstract

Standard methods for mass analysis measure ensembles of thousand to millions of molecules. This approach enables analysis of monodisperse recombinant proteins, whereas some heterogeneous protein assemblies pose a significant challenge, whereby co-occurring stoichiometries, sub-complexes, and modifications hamper analysis using native mass spectrometry. To tackle the challenges posed by mass heterogeneity, single-particle methods may come to the rescue. Recently, two such approaches have been introduced, namely, mass photometry (MP) and Orbitrap-based charge detection mass spectrometry (CDMS). Both methods assess masses of individual molecules, albeit adhering to distinct physical principles. To evaluate these methods side by side, we analyzed a set of ribosomal particles, representing polydisperse ribonucleoprotein assemblies in the MDa range. MP and CDMS provide accurate masses for intact ribosomes and enable quantitative analysis of concomitant distinct particles within each ribosome sample. Here, we discuss pros and cons of these single-molecule techniques, also in the context of other techniques used for mass analysis.

## Highlights

• Analysis of ribosomes by mass photometry and charge detection mass spectrometry
• Pros and cons of these two single-molecule mass analysis approaches are evaluated

## Graphical abstract

**Figure:**
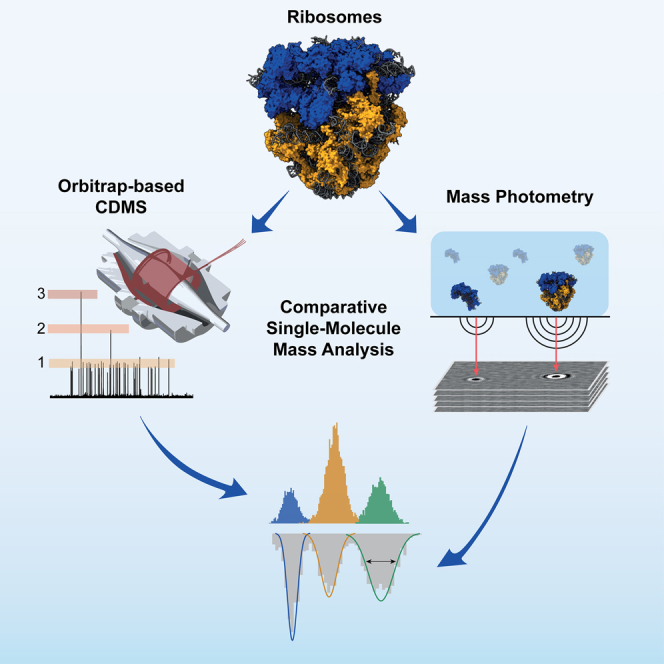

## Introduction

Traditionally, analytical approaches for mass analysis measure ensembles of thousand to millions of molecules. Such approaches work well for monodispersed, recombinantly produced proteins and protein complexes. However, assemblies from more complex eukaryotic systems especially when purified from native sources display a substantial increase in structural heterogeneity (Raynal et al., 2014), both in protein and RNA composition and stoichiometry, but especially in the presence of additional chemical post-translational modifications on either the protein or nucleic acid subunits. Among ensemble-based methods, native mass spectrometry (native MS) has emerged as a powerful tool for investigating such systems providing means for accurate mass determination of macromolecules up to several megadaltons (Leney and Heck, 2017), including intact viruses, proteasomes, and membrane protein complexes. Nevertheless, there are cases when heterogeneity of the analyzed system hampers the detection of individual charge states, which is the main prerequisite for accurate mass determination in native MS (Mckay et al., 2006). Single-molecule approaches may circumvent the shortcomings for mass analysis of highly diverse biological macromolecules.

**Figure 1:**
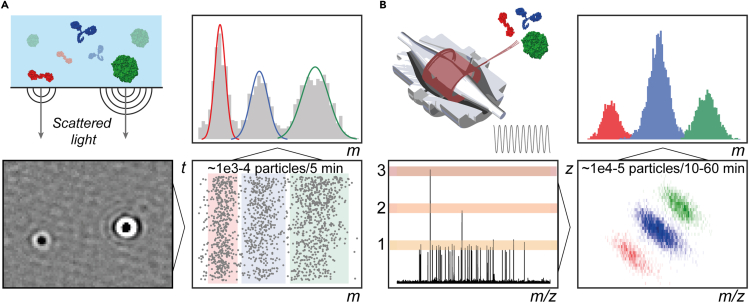
Concepts of single-particle mass analysis using mass photometry and charge-detection MS on an Orbitrap (A) In MP, biomolecules’ masses are determined through their refractivity, whereby the light scattered by a biomolecule is proportional to its mass. Upon detection of ∼2,000 individual molecules (∼5 min acquisition time), statistical analysis of the cumulative distribution of particles enables mass determination, as shown in the top-right panel. (B) In CDMS, an amplitude of the single ion signal induced on the Orbitrap electrodes is proportional to the charge of the ion. A few ion detection events are recorded per scan. After detecting ∼1e4-5 particles (∼10–60 min acquisition time), accurate masses are determined from populations of particles separated in *z* and *m/z* space. Statistical analysis of the cumulative distribution of particles enables mass determination, as shown in the top-right panel.

The recently introduced mass photometry (MP) approach is one of such methods that can analyze individual particles (Young et al., 2018) (Figure 1A). It enables accurate mass determination and quantitative analysis of distinct components in mixtures (Sonn-Segev et al., 2020; Tamara et al., 2020), and may even be used to monitor binding affinities (Soltermann et al., 2020). The method is based on principles of interference reflection and interferometric scattering microscopy, whereby the light scattering of biomolecules can be detected and directly used to extract the molecule’s mass when a sufficient number of particles have been recorded. The main advantage of such technology is its ability to analyze samples in their close to physiological environments, as it does not pose significant restrictions on the solution composition in which samples are measured. In addition, it has a relatively low bias toward specific components when mixtures are analyzed and benefits from low quantities of sample needed for mass determination. Among its limitations are the relatively large error of mass determination (2%) and dependence on the quality of the contrast-to-mass standard calibration procedure. In addition, owing to differential light scattering properties adherent to various biomolecule types, universal calibration and measurement procedures are not (yet) available. Nevertheless, MP is a powerful technique undergoing fast development and has been expanding in its application; for instance, it has been recently successfully used to assess genomic cargo loads in adeno-associated viruses (Wu et al., 2021).

Another emerging single-particle method, charge detection mass spectrometry (CDMS; Figure 1B), exhibits significant benefits in weighing macromolecular ions by simultaneously determining the charge (*z*) and mass-to-charge ratio (*m/z*) of individual ions (Keifer and Jarrold, 2017). In contrast to the conventional native MS approach, CDMS enables obtaining mass directly from these values circumventing the need for charge-resolved mass spectra. As such, this approach can overcome the challenges for analyzing highly heterogeneous biomolecular assemblies, for which—when measured by conventional native MS—individual charge states are often indistinguishable or extremely broadened, hampering accurate mass determination. Conventional CDMS approaches, including single-pass detection cylinder(s) and multi-pass ion trap-based detectors (Keifer et al., 2017), exist already for several decades and have been successfully applied to studying of various biomolecules and polymers in the wide mass ranges (Antoine, 2020; Keifer and Jarrold, 2017; Todd et al., 2020). Recently, the quantification and the appropriate calibration of induction image current for individual ions have enabled charge determination of single particles on commercially available Orbitrap mass analyzers (Kafader et al., 2020b; Wörner et al., 2020). Compared with conventional CDMS designs, charge-based mass detection in the Orbitrap mass analyzer offers more efficient single ion accumulation as well as superb *m/z* accuracy. To distinguish this approach from other types of CDMS we refer to it here as Orbitrap-based CDMS.

Unlike MP, CDMS measurement is performed in the gas phase, following ionization and desolvation of the analytes. Typically, these desolvated ionized particles carry a certain positive charge in positive ionization mode, determined by the number of charge-carrying adducts, e.g., protons. Several particles that are separated in the frequency domain (*m/z*) can be monitored individually in the same scan by the Orbitrap mass analyzer. When a sufficient number (e.g., 10^3^ to 10^5^) of single ion events is recorded, the distribution of the intact mass can be statistically deduced using each ion’s charge and *m/z*. The ability to determine mass and charge directly represents great potential for analysis of protein assemblies of increasing complexity.

Ribosomes are among the most ancient biological systems present in all living cells. They represent large ribonucleoprotein complexes responsible for protein synthesis through the translation of messenger RNA (mRNA). It has been shown that the composition and heterogeneity of ribosomes can vary significantly, not only between different organisms but even within a single cell, depending on their subcellular localization, ultimately fine-tuning gene expression (Genuth and Barna, 2018). Of note, cellular organelles of endosymbiotic origin depict unique ribosome compositions containing distinct proteins and rRNA absent in cytosolic ribosomes. The most prominent examples include the mitochondrial 55S ribosome (Kummer et al., 2018) in eukaryotic cells and the chloroplast 70S ribosome (Bieri et al., 2017) in the cells of plants. As for the complexity of cytosolic ribosomes, they have been increasing in size throughout evolution, culminating in particularly large 80S ribosomes (∼4 MDa) observed in mammalian cells, which significantly outweigh their 70S prokaryotic counterparts (∼2.5 MDa). Although the key functional elements of ribosomes remain well conserved, many species-specific and compartment-specific components have been reported, often present at substoichiometric levels (Simsek et al., 2017). Ribosome composition has also been shown to vary under the influence of environmental stimuli (Samir et al., 2018). In addition, ribosomal proteins and RNA can carry various post-translational modifications, including ubiquitination (Juszkiewicz and Hegde, 2017), phosphorylation (Ruvinsky and Meyuhas, 2006), methylation (Chang and Chang, 1975) on ribosomal proteins, and RNA modifications (Sloan et al., 2017). Furthermore, some molecules of pathogenic origin tend to bind to host ribosomes to impose the production of exogenous proteins. For example, the hepatitis C virus internal ribosome entry site (HCV IRES) RNA binds explicitly to the human small 40S ribosome (Spahn, 2001), further extending the pool of potential interacting partners. Such compositional variability and the presence of both protein and RNA components and their various modifications make ribosomes extremely challenging for accurate mass analysis.

Recently, through significant advances in structural biology, primarily in cryo-electron microscopy (cryo-EM), the complicated biogenesis and maturation pathways of ribosomes have been unraveled for eukaryotic organisms (Baßler and Hurt, 2019; Klinge and Woolford, 2019). However, for many other organisms, the details and landscape of ribosomal assemblies remain still understudied. Furthermore, it is now becoming more evident that mass analysis of ribosomes through native MS or other mass analysis techniques, such as light scattering methods (Minton, 2016) or analytical ultracentrifugation (Berkowitz and Philo, 2020), can shed light on sample diversity, providing information complementary to high-resolution structural methods. Although ribosomes have been successfully measured with native MS revealing multiple mass variants for bacterial ribosomes (Mckay et al., 2006; Van De Waterbeemd et al., 2017), for the more complex eukaryotic ribosomes, native MS experiments available in the literature are limited to the small 40S ribosomal particles (Van De Waterbeemd et al., 2018), mainly due to its relative simplicity, i.e., containing a single 18S rRNA molecule and 33 ribosomal proteins. The more complex large 60S and intact 80S eukaryotic ribosomes contain multiple rRNA molecules and a larger number of subunits and potential binders. Moreover, native MS measurements of such systems are further exacerbated by their dependence on high salt concentrations, e.g., Mg^2+^ ions are necessary for stabilizing the protein-rRNA interfaces (Moore and Spremulli, 1985), leading to extensive adduct formation, which becomes more detrimental with increasing mass of the analyte (Mckay et al., 2006). Therefore, intact ribosomes and many other large endogenous machineries remain highly challenging for conventional mass analysis, and, therefore, the emergence of new approaches is desired.

Here, we describe, evaluate, and compare two emerging single-particle technologies, MP and Orbitrap-based CDMS (Figure 1), for analysis of ribosomes from various origins (Table 1), including the intact 80S ribosome from human cells (Hs80S) and 70S chloroplast ribosome from spinach (So70S). We demonstrate how both methods enable the measurement of such large and heterogeneous biological systems. First, we benchmark the methods for analyzing a relatively homogeneous human small 40S ribosome (Hs40S), demonstrating a complete agreement with prior high-resolution native MS analysis. We then proceed to analyze the larger and more complex So70S and Hs80S samples, whereby small, large, and intact ribosomal particles are measured simultaneously. These samples are highly challenging for mass analysis using conventional native MS. In a final test case, we explore resolution limits of the two single-particle methods by focusing on the Hs40S particle when bound to the viral HCV IRES RNA, which upon binding increases the mass of the particles by ∼8%. A comparison of the two techniques and the prospects of MP and CDMS within the mass analysis toolbox are provided in the final part.

**Table 1:** Overview of the ribosomal samples analyzed by MP and CDMS

	Hs40S	Hs60S	Hs80S	So30S	So50S	So70S
Organism	*H. sapiens*	*H. sapiens*	*H. sapiens*	*S. oleracea*	*S. oleracea*	*S. oleracea*
Subcellular location	Cytosol	Cytosol	Cytosol	Chloroplast	Chloroplast	Chloroplast
Number of proteins	34	47	81	25	34	59
rRNA fragments	18S (HCV IRES RNA)	28S, 5S, 5.8S	18S, 28S, 5S, 5.8S	16S	23S, 4.5S, 5S	16S, 23S, 4.5S, 5S
Theoretical mass (kDa)	1,214 (1,317)^b^	2,633	3,847	909	1,539	2,448
Mass from Native MS (kDa)	1,220 (1,324)^b^	2,585	–a	861	1,456	2,353

See also Figures S1 and S3, and Tables S1–S4.

a Could not be measured by standard native MS, but a mass of 3,805 kDa is suggested by summing up the Hs40S and Hs60S masses.

b The mass in parenthesis is that of the IRES-bound Hs40S ribosomal particles.

## Results and discussion

### Analysis of human 40S ribosomal particles with single-particle MP and Orbitrap-based CDMS

**Figure 2:**
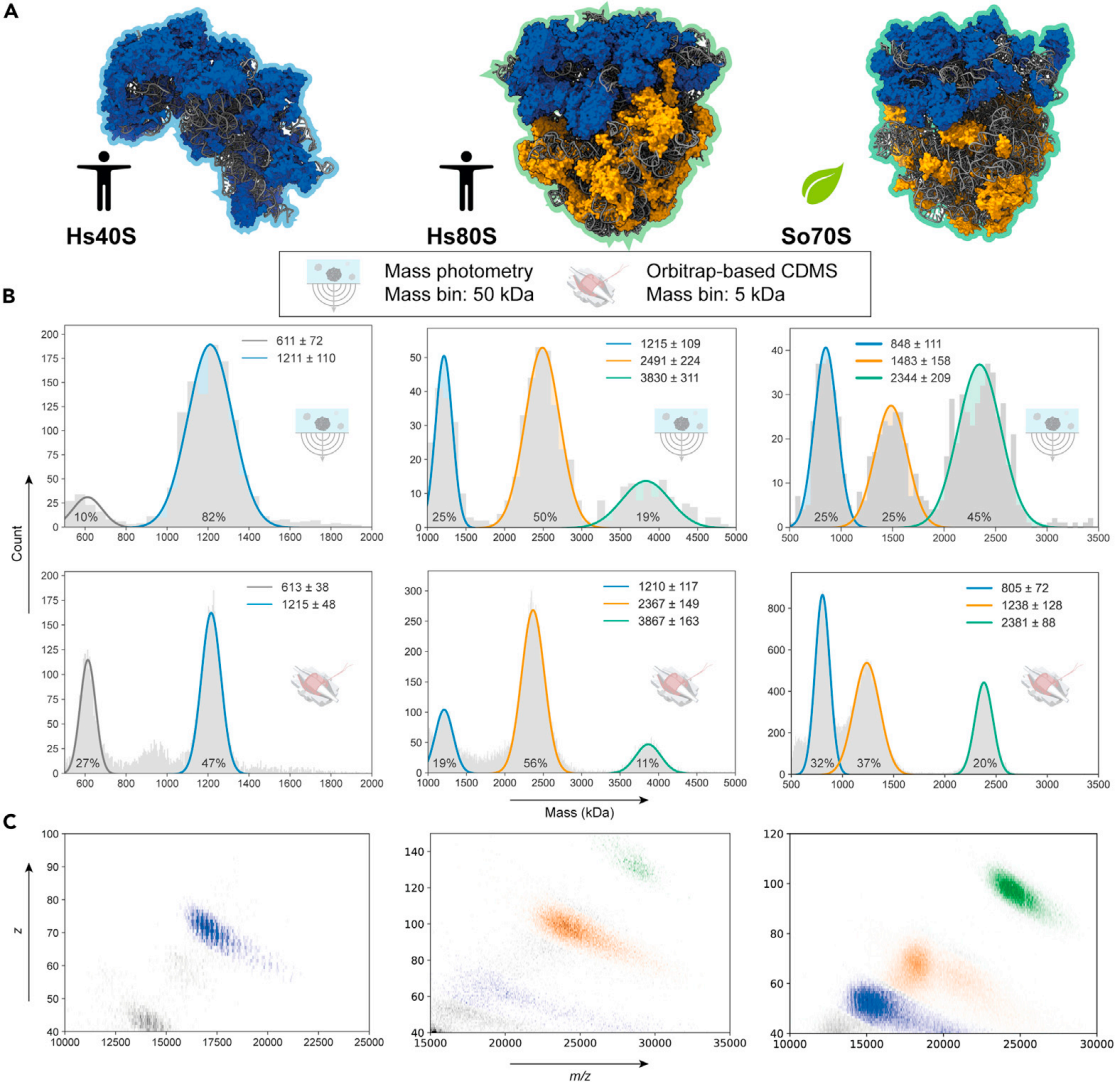
Single-particle analysis of intact ribosomal particles by MP and Orbitrap-based CDMS (A) Cartoons depicting the structures of the Hs40S (left; PDB: 5A2Q), Hs80S (center; PDB: 4UJC), and So70S (right; PDB: 5MMM) ribosomes. (B) Mass distribution histograms obtained for the Hs40S (left), HS80S (center), and So70S (right) ribosomal particles using MP (top) and CDMS (bottom). The fitted mean mass and standard deviation of the most abundant species are provided, using the same color coding for MP and CDMS data. Normalized counts (%) for each detected species in the displayed mass ranges are indicated within each fitted normal distribution. See also Figure S2 showing single-molecule mass analysis of Hs60S and Table S5 containing technical details for each measurement visualized in (B). (C) 2D histogram representations of the CDMS data with color-coded and annotated co-occurring particle detection events, showing the improved separation of distinct ribosomes achieved by using both the *z* and *m/z* dimensions.

To benchmark the performance of the two single-particle approaches, we initially analyzed human small 40S ribosome particles. In Figure 2B mass distribution histograms (left column) are shown for the analysis of the Hs40S particles obtained by MP and CDMS. These Hs40S samples provide an ideal benchmark as they were previously analyzed by high-resolution native MS by Van de Waterbeemd et al. (2018) using an Orbitrap UHMR, revealing their high purity and uniformity. Hs40S represents a ribonucleoprotein complex comprising 33 ribosomal proteins and a single 18S rRNA molecule. Following processing of single-particle data, the fitted mean mass and standard deviation of the most abundant species (blue traces) in the mass distributions were 1,211 ± 110 and 1,215 ± 48 kDa for MP and CDMS, respectively. Although the mass distribution in the MP measurement is about twice broader than that observed by CDMS, likely due to the lower resolving power of the MP instrument, the fitted mean mass by both techniques is very close to the theoretical mass of intact Hs40S ribosome (1214 kDa), which was estimated from the protein sequences and subunit masses previously determined by top-down LC-MS/MS analysis (Van De Waterbeemd et al., 2018) and the theoretical mass of the S18 rRNA (Table 1; also see Table S1 for a complete list of involved protein and RNA subunits). The observed masses are also in excellent agreement with the mass detected via high-resolution native MS (1,220 kDa) based on charge state assignment strategy (Figure S1), which was possible due to the relatively monodisperse nature of the Hs40S particles and the advanced desolvation achievable on the Orbitrap platform. Of note, signals from distinct charge states of the intact Hs40S particle are also present in the 2D histogram (*m/z* and *z*) representation of the current Orbitrap-based CDMS data (Figure 2C; left panel). In the mass histograms of the Hs40S preparation, additional particles are observed of lower abundance (gray trace), with an apparent mean mass of ∼610 kDa by both MP and CDMS. These particles likely represent co-purified subcomplexes of the large 39S mitochondrial ribosome, for which several proteins were identified in the bottom-up MS analysis at abundances slightly lower than those of Hs40S proteins (Van De Waterbeemd et al., 2018). Although in MP, Hs40S accounted for more than 80% of detected particles in the range from 500 to 2000 kDa, Orbitrap-based CDMS demonstrated more particles of lower mass contaminant, with only ∼50% of particles attributed to Hs40S (Figure 2B). This discrepancy likely stems from challenges associated with effectively ionizing, transmitting, and detecting large ribonucleoprotein assemblies in vacuum-based Orbitrap mass analysis.

In summary, the data obtained for the Hs40S particles by MP and Orbitrap-based CDMS are in good agreement with previous data obtained for the mass analysis by high-resolution native MS (Van De Waterbeemd et al., 2018). Both MP and Orbitrap-based CDMS can determine an accurate mass of these 1.2-MDa ribonucleoprotein assemblies, providing masses within 0.2% of the expected theoretical values. Even though 50% of the Hs40S mass is comprised by 18S rRNA, MP still yielded an accurate mass of the ribosomal particles using a standard protein-based calibration mixture. This result hints at refractive properties of globular ribonucleoproteins that are comparable with those of fully proteinaceous molecules. Our observations are to an extent supported by Li et al. who observed that proteins and single-stranded DNA (ssDNA) have more comparable contrast-to-mass ratios than ssDNA and double-stranded DNA (Li et al., 2020), which might be influenced by the differences in polarizability of these molecules. We further reinforce these conclusions by comparing contrast-to-mass of intact cytosolic and chloroplast ribosomes with fully protein-based particles of comparable mass below.

### Single-particle mass analysis of eukaryotic 80S and chloroplast 70S ribosomes

Motivated by extending the single-particle analysis to larger and more complex systems, we next measured mass distributions of human 80S and chloroplast 70S ribosomes using both MP and CDMS. The human cytosolic 80S ribosome is built up from the smaller 40S subunit and the larger 60S subunit, whereas the chloroplast 70S ribosome is built up from the 30S and 50S subunits. Distinctly from the cytosolic Hs80S ribosome, chloroplast ribosomes comprise proteins synthesized in the chloroplast and the nucleus, with the latter requiring transit peptides and specific modifications to get efficiently transported into the chloroplast (Bruce, 2000). We previously determined that in the chloroplast ribosomes many protein subunits are represented by a variety of isoforms (i.e., distinct amino acid sequences), which further increases the heterogeneity of chloroplast ribosomes (Figure S3) (Van De Waterbeemd et al., 2018).

Both human 80S and chloroplast 70S ribosomes require a substantial amount of Mg^2+^ ions to prevent dissociation into the small and large subunits (Nierhaus, 2014). In this work, we opted for a concentration of 2–5 mM of Mg^2+^, which seemed ideal for detecting intact ribosomes, although we also observed some residual smaller subunits. The required addition of Mg^2+^ ions inevitably leads to an additional broadening of the mass distribution, hampering further the high-resolution mass analysis by standard native MS, whereby charge-resolved spectra are essential. In single-particle approaches the incorporation of Mg^2+^ by ribosomes does not hamper the mass analysis since the mass of each particle is determined individually.

In Figure 2B, mass distribution histograms are shown for the analysis of the cytosolic Hs80S ribosome (middle column) and the chloroplast 70S ribosome (right column) particles performed by MP and Orbitrap-based CDMS, respectively. For the purified Hs80S sample, three major mass distributions were observed both by MP (with the masses of 1,215 ± 109, 2,491 ± 224, and 3,830 ± 311 kDa) and Orbitrap-based CDMS (with the masses of 1,210 ± 117, 2,367 ± 149, and 3,867 ± 163 kDa), respectively (Figures 2A–2C, center panels). These three distinct particle distributions were assigned as representing the Hs40S, Hs60S, and Hs80S ribosomal particles. Except for the Hs60S subunit, the measured mean masses by both MP and CDMS were found to be very close to the theoretically expected values with a deviation of less than 1%. This is also the first report of accurately measuring intact Hs80S ribosomes using single-particle mass photometry and mass spectrometry analysis, which is not easily manageable by conventional native MS. For Orbitrap-based CDMS, an elevated higher-energy collisional dissociation (HCD) voltage (200–250 V) was required for detecting the very large ∼4-MDa intact Hs80S particles, and as a consequence, some dissociation of the concomitant small and large ribosomal subunits was observed. This gas-phase dissociation causes a tail in the mass distribution as shown in the 2D histogram view with the charge-reduced dissociated particles appearing at higher *m/z*. Harsher conditions used in gas-phase-based analysis, as compared with solution-based MP, likely underlie diminished relative abundance of intact Hs80S ribosome in Orbitrap-based CDMS (Figure 2B, middle panels). Furthermore, the mass distribution of the Hs40S ribosome displays in these data a full width at half maximum (FWHM) nearly 2.5 times wider than in the case when the separately purified Hs40S is measured (left column). This broad distribution may imply a higher heterogeneity of Hs40S when it is in equilibrium between the detached and Hs60S-bound forms, and/or be the consequence of the higher concentration of Mg^2+^ used.

Pleasingly, the mass of the intact Hs80S ribosomes detected by MP at 3.85 MDa here is close to the theoretically expected mass, based on the masses of the protein and RNA subunits (Tables 1, S1, and S2). Also, the mass of the Hs40S detected in these analyses (1.2 MDa) is spot-on. However, the measured mass of the putative Hs60S ribosomal particles is lower than theoretically expected (∼2.63 MDa). One possible explanation lies in a relatively high RNA-to-protein ratio of Hs60S (∼1.9:1) compared with Hs40S (∼1:1), making Hs60S more dependent on Mg^2+^ or binding to Hs40S for stabilization. Besides, the lower mass of the Hs60S ribosome particles could also originate from partial loss of the stalk proteins that are known to dissociate easily (Davydov et al., 2013). To determine whether a more accurate mass of Hs60S could be obtained, we also performed MP and Orbitrap-based CDMS of the intact Hs60S ribosome, purified separately (Figure S2). Of interest, with MP we obtained a mass of 2.58 MDa, which was extremely close to the mass determined with conventional native MS (2.58 MDa; Figure S1C), although the native mass spectrum is of low quality and seems to represent multiple superimposed charge distributions. In Orbitrap-based CDMS, when measuring the separately purified Hs60S, multiple putative mass peaks could be fitted into a rather broad mass distribution, with the major species (2.61 MDa) in the distribution close to the theoretical value and to the masses obtained by other approaches. Consistent determination of ∼2.6 MDa mass when Hs60S is purified separately indicates that the Hs60S can be retained more intact when there is no exchange between the Hs40S-bound and free forms.

Similarly to the analysis of the Hs80S sample, three major mass distributions were observed for the chloroplast 70S ribosomes corresponding to So30S, So50S, and intact So70S particles (Figures 2A–2C, right panels). Unlike the Hs40S and Hs60S subunits, where native MS measurements have previously provided masses very close to theoretical mass (Figure S1), for chloroplast ribosomes, native MS yielded values substantially lower (∼3%–5%) than expected for the So30S, So50S, and So70S particles (Table 1; see also Figure S3 for standard high-resolution native MS data and Tables S3 and S4 for experimental masses of ribosomal subunits). This implies either an overestimation of theoretical mass or a possible solution-phase dissociation of the ribosomal particles. The measured mean masses of the So30S and So50S ribosomes obtained with MP (848 and 1,483 kDa, respectively) are close to the masses measured by high-resolution native MS (861 and 1,456 kDa; Figure S3), albeit distinct from the (sequence-based) theoretical masses (909 and 1,539 kDa). The slightly lower mass obtained by MP when compared with native MS could potentially be attributed to the 1,000x drop in concentration (5 nM and 5 μM, respectively). As for CDMS, both small and large subunits demonstrate somewhat lower masses than in native MS, with the mass of So50S particles having the largest mass difference, similar to the observations made for the Hs60S subunits when analyzing the Hs80S sample. Nevertheless, in both MP and Orbitrap-based CDMS, the mean mass of the intact So70S (2,344 and 2,381 kDa, respectively) remains in better agreement with the native MS result (2353 kDa) compared with the theoretical mass (2448 kDa). Therefore, the mass of chloroplast 70S ribosomes obtained by native MS is more convincing as the reference mass for both single-particle measurements than the sequence-based theoretical mass. Finally, similar to the Hs80S sample, intact So70S has been detected at lower relative abundance in Orbitrap-based CDMS compared with MP (Figure 2B, right panels).

### Monitoring the binding of HCV IRES RNA to Hs40S

As demonstrated in the previous sections, MP and CDMS provide alike mean mass values, but CDMS exhibits in general higher resolution than MP, especially for very large complexes. Although the resolution obtained by CDMS is still lower than that attainable by conventional high-resolution native MS, the extra *z-*dimension in CDMS provides additional opportunities for resolving close-in-mass species. To test this unique feature, we analyzed the human 40S ribosome incubated with varying concentrations of the internal ribosome entry site (IRES) RNA element from hepatitis C virus (HCV).

**Figure 3:**
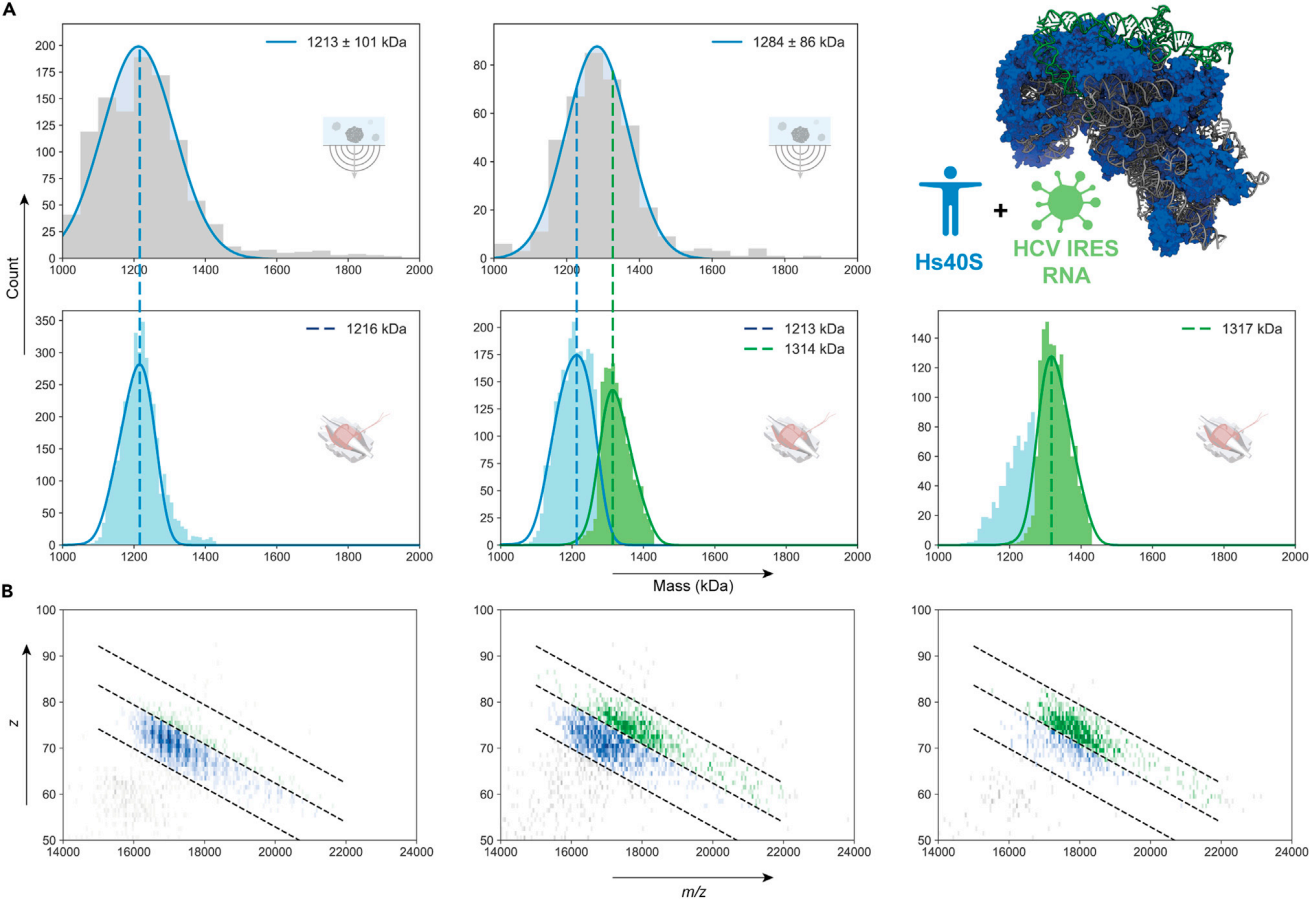
Monitoring the binding of HCV IRES RNA to Hs40S using single-particle approaches (A) Mass distribution histograms obtained using MP (top) and CDMS (bottom) for the Hs40S alone (left) and when incubated with either low (center) or high (right) concentration of HCV IRES RNA. In the top right, the cartoon shows the Hs40S ribosomal particle (blue and gray) bound to HCV IRES RNA (green) (PDB: 5A2Q). (B) 2D histogram representations of the CDMS data from (A) illustrating the additional separation in the *z*-space. See also Table S5.

A transformation process from free Hs40S ribosome particles to particles fully containing Hs40S and HCV IRES at a 1:1 stoichiometry could be observed (Figure 3). There is a noticeable increase of 101 kDa (from 1,216 to 1,317 kDa) in mass upon addition of HCV IRES RNA, which agrees with the free HCV IRES RNA mass (103.7 kDa) as measured by the conventional high-resolution native MS. In addition, we monitored an intermediate state with a mixture of IRES-bound and free Hs40S by adding HCV IRES RNA at lower concentrations (Figure 3A, middle column). For MP, a single distribution with a mean mass of 1,284 kDa is observed. The large FWHM of ∼100 kDa obtained by MP substantially hampers the detection of distinct IRES-bound and -unbound forms. For CDMS, the two distributions also overlap (Figure 3A; bottom center panel), albeit that the additional *z*-dimension enables separating these two distributions (Figure 3B). In this manner, the mass distributions of the two individual species can be separated in the mixture according to the boundaries of the free and IRES-bound Hs40S. Subsequent data processing of the isolated populations of these particles provided masses of 1,213 and 1,314 kDa, which are highly consistent with the expected mass of free and IRES-bound Hs40S. Overall, although CDMS cannot directly disentangle the close-in-mass populations of these particles, its extra *z*-dimension provides means for separation, enabling not only monitoring the formation of complexes but also validating the expected 1:1 stoichiometry of HCV IRES RNA binding to the Hs40S ribosome. MP, on the other hand, can only hint at the formation of this complex by displaying a uniform shift in mass of the entire particle distribution.

### Exploring performance metrics in single-particle mass analysis

**Figure 4:**
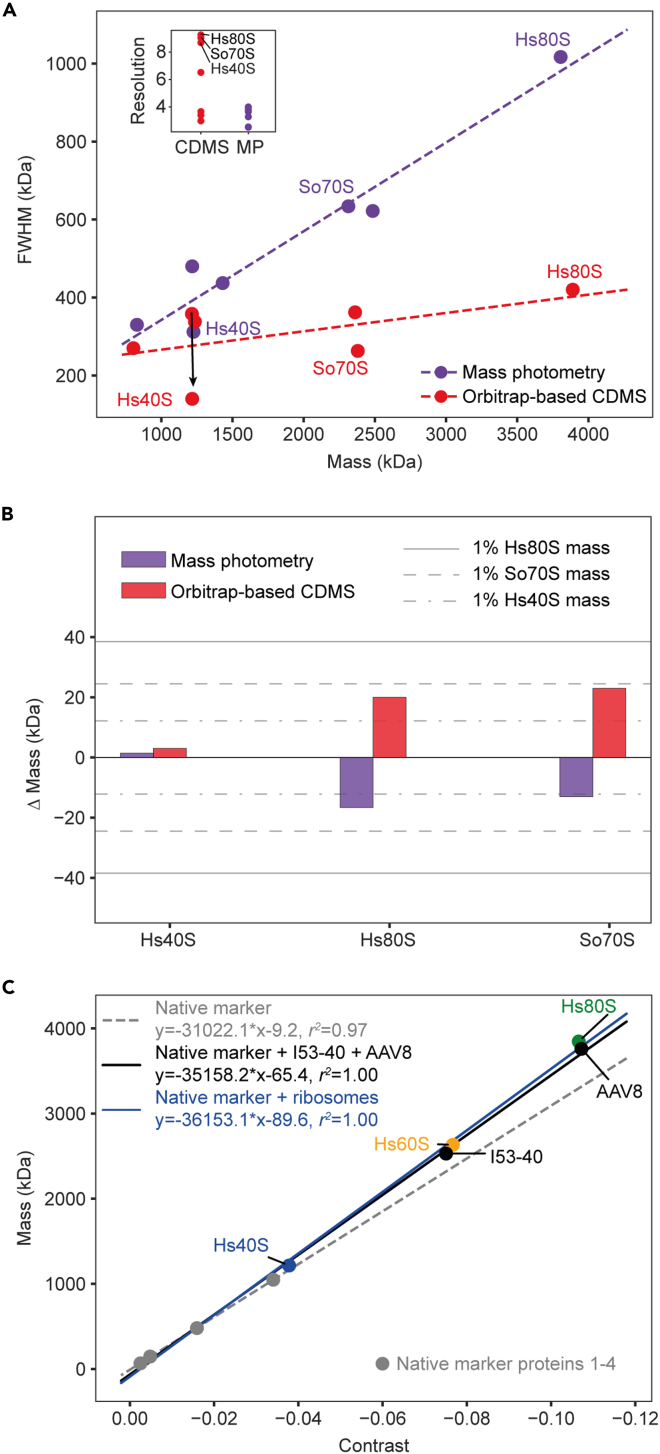
Metrics of performance in CDMS and MP (A) Experimental peak width (expressed as FWHM) for all measured ribosomal particles with CDMS (red dots) and MP (purple dots) reveals that CDMS achieves substantially higher resolution, especially for the larger particles analyzed (inset). See also Table S5. (B) Mass accuracy determined by CDMS and MP for Hs40S, Hs80S, and So70S ribosomal particles. The dash-dot-dash, dashed, and solid lines provide the 1% mass threshold for the Hs40S, So70S, and Hs80S particles, respectively. (C) The contrast-to-mass calibration curves produced for mass determination with MP either using the manufacturer-recommended native marker protein standard (dashed gray line) alone or extended with either in-house analyzed MDa proteinaceous particles (I53-40 nanoparticles and adeno-associated virus AAV8 particles, solid black line) or human ribosomal particles (Hs40S, Hs60S, and Hs80S, solid blue line).

Mass resolution and mass accuracy are some of the crucial measures of performance in mass analysis. Here, we applied single-particle techniques for measuring the mass distribution of various intact ribosomal particles. Resolution for MP and CDMS can be defined as *m*/*Δm* for the detected distributions of the particles, where *m* is the mean mass of distribution and *Δm* is its FWHM. Our analysis shows that the FWHM for particles in the megadalton range detected with CDMS is substantially lower than in the case of MP (Figure 4A). The attainable resolution in CDMS is consistently about 9–11 for the intact ribosomal particles (Figure 4A, inset). However, for the smaller constituents of Hs80S and So70S samples, namely, Hs40S, Hs60S, So30S, and So50S ribosomal particles, the resolution is further decreased owing to peak broadening by additional heterogeneity. This may be caused by the gas-phase dissociation and ion signal instability at the conditions favoring detection of large intact ribosomes, as specific sample components may require different transmission and ion cooling parameters for optimal detection. Indeed, when the purified Hs40S particles are measured separately at lower collisional activation energies, the FWHM and resolution become substantially improved (Figure 4A, black arrow). Of interest, when activation energies are further increased for Hs40S subunits, we do not observe significant broadening of the peak, so the exact reasons behind the poor resolution of Hs40S subunits in mixed populations of ribosomal particles remain elusive.

On the contrary, the FWHM measured with MP grows linearly with the increasing mass of the analyzed ribosomal particles (Figure 4A; *r*^2^ = 0.94), which is primarily dictated by the resolving power limits of the instrument that previously have been attributed to the photon shot noise, which scales linearly with mass (Young et al., 2018). In contrast with CDMS, MP does not demonstrate any bias toward specific components in the mixture, with all ribosomal particles displaying a resolution of ∼3–4. As a consequence of this lower resolution, an appropriate bin size selection is critical for processing MP data, especially for Gaussian fitting, whereby small window binning may result in the splitting of broad mass distributions and potential overinterpretation of data. In this study, we first performed a kernel density estimation of the data to predict the maxima and FWHM and then selected a 50 kDa bin optimal for Gaussian fitting and mean mass determination (see STAR Methods for further information on our data processing). In summary, although instrumental limits drive the broad mass distributions observed for intact ribosomal particles by MP, the resolution attainable with CDMS is significantly less dependent on instrumental limits and is more reflective of the sample’s intrinsic heterogeneity. Interpretation of Orbitrap-based CDMS data, however, should take into account instrumental conditions, as they can bias mass detection for specific components in mixtures of analytes covering a wide mass range.

Mass accuracy is another important metric of mass analysis, which significantly depends on the attainable mass resolution and, most notably, mass calibration. Mass calibration in CDMS consists of two parts. First, *m/z* is calibrated with high-concentration CsI clusters, resulting in less than 50 ppm mass accuracy even for large protein assemblies (e.g., 20 ppm for 800 kDa GroEL) (Rose et al., 2012). Second, intensity-to-charge calibration of the Orbitrap mass analyzer is performed, which as earlier shown, provides a root-mean-square deviation (RMSD) of 3.5 elementary charges in a wide mass range of 0.2–9 MDa (Wörner et al., 2020). The intensity-to-charge calibration ultimately defines the attainable mass accuracy, resulting in considerable mass error within 2%. Favorably, the intensity-to-charge calibration has shown exceptional robustness for a given Orbitrap instrument, with measurements made 3 months apart providing a nearly identical calibration (Wörner et al., 2020). Moreover, by averaging over a large number of single particles in a typical Orbitrap-based CDMS experiment (∼1e4-5), it is possible to reach relatively high mass accuracy within 1% as also demonstrated here (Figure 4B).

In the case of MP, a contrast-to-mass calibration is typically performed using several proteins and protein complexes of known mass (Young et al., 2018), providing mass accuracy of approximately 2%, which is quite close to the theoretical mass error observed for Orbitrap-based CDMS (Figure S3). However, the accuracy of the mass measurement in MP is quite sensitive to the quality of the mass calibration, which is done relatively often owing to minor changes in the optical alignment of the instrument. Using a native marker protein standard for contrast-to-mass calibration in the range below 1 MDa, we were readily able to determine masses of intact ribosomal particles within 1% (Figure 4B), despite their high RNA content (50%–60% of total mass). Notwithstanding, for intact ribosomes we observed consistently lower masses by using MP for the larger intact ribosomes than in Orbitrap-based CDMS. Although the MP vendor recommends use of a DNA-based calibrating mixture for accurate mass determination of nucleic acids, it has been shown that contrast-to-mass ratios are not so different between proteins and ssDNA (Li et al., 2020). Therefore, we hypothesize here that, when rRNA molecules are intertwined with proteins forming globular macromolecules, their refractive properties are comparable with those of fully proteinaceous species. To reinforce this, we extended the calibration curve using fully proteinaceous particles of known mass, 2.53-MDa I53-40 nanoparticles and 3.73-MDa empty capsids of adeno-associated virus (AAV8) (Figure 4C). Of note, the calibration curve extended with the ribosomal particles is similar to that produced by using fully proteinaceous particles, indicating little difference in refractivity between these macromolecules (Figure 4C, blue and black lines, respectively). Nevertheless, the extended mass range calibration shows better suitability for accurate mass analysis of MDa particles and therefore should be considered for accurate mass analysis of large species in the MDa range.

Overall, in this work, MP and CDMS enabled the detection of intact ribosomes with a mass accuracy within 1%, demonstrating excellent compatibility for analysis of large heterogeneous assemblies, which may be challenging when analyzed by conventional high-resolution native MS. Also, see Table S5 for additional metrics behind the here-presented single-particle measurements, including quantification of each ribosomal species within the samples.

### Comparison of single-molecule methods with conventional high-resolution native MS

Given the distinct physical principles of mass assessment, MP and Orbitrap-based CDMS can be highly complementary. Here, we attempt to compare the pros and cons of these two emerging single-particle mass analysis methods and use the more conventional high-resolution native MS approach as a reference (Table 2).

**Table 2:** An overview of strengths and weaknesses for different mass analytical approaches

	MP	Orbitrap-based CDMS	Conventional native MS
Environment	Solution phase	Gas phase	Gas phase
“Nativeness”	+++	+	+
Typical acquisition time	∼5 min	∼10–60 min	∼5–15 min
Processing software	Commercial	Custom scripts	Commercial
Sample consumption	1–10 nM	10 nM–1 μM	1–10 μM
Sample volume	1–10 μL	1–10 μL	1–10 μL
Resolution	∼4	∼10	∼100–200^a^
Accuracy	<2%	<2%	<1%^b^
Typical molecule count	1e3-4	1e4-5^c^	>1e6
Max reported mass	∼5 MDa^d^	∼10 MDa	∼10 MDa
Detection limitations	<50 kDa, low K_d_	z < 10+, buffer	High heterogeneity, buffer
Dynamic range	+	++	+++
Cost	$	$$$	$$$

a Based on the data from Figures S1 and S3.

b Requires charge-state separation and is based on Van De Waterbeemd et al. (2017).

c After data filtering.

d Reported by the vendor.

One of the major advantages of MP compared with the MS-based methods is that MP is a solution-phase-based method, whereby virtually no sample preparation is needed, apart from reaching the desired concentration, typically achieved through rapid dilution in an appropriate buffer. This enables maintaining a native environment during sample acquisition. In contrast, MS-based methods probe macromolecular ions in the gas phase, which, albeit has been shown to allow retention of native-like features, implies significant limitations for specific biological systems, notably also ribosomes, that contain a relatively large amount of negatively charged RNA and require high ionic strengths and the presence of MS-unfriendly salts, e.g., Mg^2+^. To facilitate mass analysis with native MS and CDMS, it is often required to buffer exchange the sample into a solution containing only volatile salts, such as ammonium acetate, which has been proven extremely useful for analyzing various macromolecules owing to its ability to mimic to some extent the physiological environment (Konermann, 2017).

The necessary ionization and desolvation of the molecules prior to MS analysis is achieved through ion manipulation under relatively harsh conditions, typically using high voltages and strong electrical or magnetic fields to transmit and desolvate the ions en route to the mass analyzer. Such handling can often induce sample deterioration, which potentially results in erroneous mass analysis. In contrast, MP does not require additional potentially destructive forces to be applied to the sample except for a low-power laser, reducing the possible degradation of the analyte. Indeed, for mass analysis of the intact Hs80S and So70S ribosomes by MS, high activation energies were needed to optimize ion transmission, cooling, and detection. Consequently, we observed lower resolution for concomitant lower mass species than for intact ribosomes (Figure 4A). In contrast, all co-occurring species were detected without noticeable bias in the MP analysis. Furthermore, the acquisition time for a typical MP experiment is short (∼5 min/sample), accompanied by a very low sample consumption with the optimal concentration range being 1–10 nM. As for Orbitrap-based CDMS, although it features a longer acquisition time of about 10–60 min to accumulate sufficient single-particle events for statistical analysis, it also requires a relatively low amount of sample, e.g., 100 nM, which is an order of magnitude lower than the concentration typically used in conventional native MS (0.5–10 μM).

Considering eases of use and sample consumption, MP seems to be the favorable method of choice. However, its resolution and accuracy of mass determination fall short of what is possible with MS-based approaches. Native MS as the most mature and established mass analytical technique outperforms both CDMS and MP in mass accuracy and resolution, providing parts per million mass errors for large protein assemblies and resolution limited only by the sample purity (Lössl et al., 2014). However, highly heterogeneous samples can substantially distort mass analysis with standard native MS owing to the broadening of overlapping charge states induced by the presence of a multitude of small mass modifications, overlapping oligomeric states, incomplete desolvation, or combinations thereof. Both MP and CDMS approaches largely circumvent these issues.

Here, we demonstrated that CDMS provides a better resolution and statistics of mass determination than MP. This is primarily achieved through a higher number of detected particles per typical experiment and reduced intrinsic mass error, enabled by a more precise and robust charge-determined calibration. Although the slightly reduced resolution obtained by MP depends heavily on the quality of the contrast-to-mass calibration, both methods eventually provide similar accuracy of less than 1% mass error for the here-analyzed ribosomal particles. CDMS can enable two-dimensional separation in *m/z* and *z*-space, which provides additional means for data analysis, boosting accuracy and resolution and potentially separating overlapping signals in one of these dimensions.

**Figure 5:**
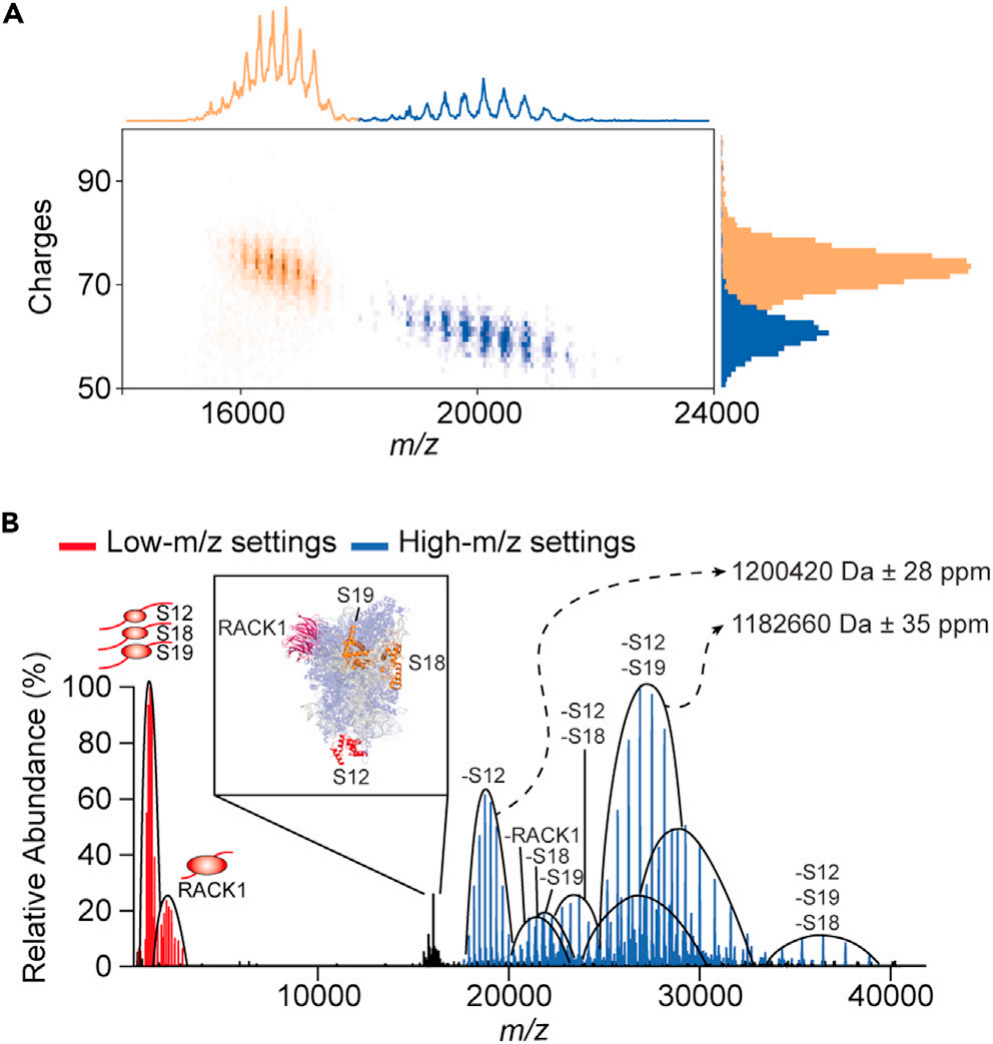
Native top-down analysis of Hs40S by CDMS and conventional native MS (A) Detection of dissociation products of Hs40S in tandem MS analysis, by using CDMS. Intact Hs40S ribosomal particles (i.e., the precursor ions) and particles missing the S12 ribosomal protein (the fragment ions) are displayed in orange and blue, respectively, in all three representations, including a 2D histogram, a cumulative *m/z* spectrum, and the charge histogram. Precursor and fragment ions are clearly separated in *m/z* and *z* dimensions. (B) Native top-down analysis of dissociation products of Hs40S by conventional high-resolution native MS is shown for comparison with CDMS. A composite spectrum is shown displaying low-*m/z* (red) and high-*m/z* (blue) fragment ions following the isolation and fragmentation of the most abundant precursor (black) charge state of Hs40S. The inset shows the structure of the Hs40S with dissociating peripheral subunits highlighted in red, orange, and pink. Charge distributions corresponding to the different dissociating products are indicated by solid black lines with the masses shown for the two most abundant high-*m/z* species. The collisional energies applied in (B) are higher than in (A), and, therefore, subsequent losses of multiple subunits are observed in (B) (e.g., loss of S12 and S19 ribosomal proteins).

Finally, we would like to point out that despite the great potential of mass analysis using single-particle approaches, conventional native MS remains the most powerful and versatile tool at the moment. The excellent ability to combine different tears of ensemble-based MS approaches with native MS has been highlighted in a recent review (Wu and Robinson, 2021). Furthermore, native top-down MS, where macromolecules are interrogated not only when intact but also upon gas-phase activation, has enabled extracting information beyond the intact mass analysis. High-resolution mass analysis of the various dissociation products can help to determine the composition, stoichiometries, and binding stabilities. Although CDMS potentially can also use these features, it still requires further optimization. To exemplify this, in Figure 5, we show and compare the application of Orbitrap-based CDMS and conventional native MS for the native top-down analysis of Hs40S ribosome particles. Although CDMS readily provides charge- and *m/z*-resolution of a single dissociation product in the high-*m/z* range, native MS resolves and allows the mass analysis of multiple co-occurring dissociation products with outstanding mass accuracy and resolution. Orbitrap-based CDMS application to conventional top-down MS has already shown significant advances in analyzing complex protein mixtures without chromatographic separation (Kafader et al., 2020a). Therefore, the extension of CDMS for superior native top-down MS analysis is not far off.

### Limitations of the study

Two methods used in this work interrogate analytes in different environments. For MP, analytes are analyzed in solution, whereas Orbitrap-based CDMS is focused on studying molecules upon transfer into the gas phase via nano-electrospray ionization. Besides, a buffer exchange is required for Orbitrap-based CDMS measurements. These differences may result in method-specific biases that may affect quantification and detection of certain macromolecules. In addition, differential refractive properties of protein and RNA molecules might contribute to an error for mass determination with MP, although it has been shown that contrast-to-mass ratios are comparable between single-stranded DNA and proteins (Li et al., 2020). Additional studies have to be conducted to determine a more optimal calibration solution for ribonucleoprotein molecules.

## STAR★Methods

### Key resources table

**Table:** 

REAGENT or RESOURCE	SOURCE	IDENTIFIER
**Biological samples**		

Human 40S ribosomal particles	Nenad Ban group (ETH Zurich)	(Weisser et al., 2017)
Human 60S ribosomal particles	Nenad Ban group (ETH Zurich)	(Weisser et al., 2017)
Human 80S ribosomal particles	Nenad Ban group (ETH Zurich)	(Quade et al., 2015)
Chloroplastic 70S ribosomal particles from Spinach	Nenad Ban group (ETH Zurich)	(Bieri et al., 2017)

**Chemicals, peptides, and recombinant proteins**		

I53-40 nanoparticles	Neil P. King group (University of Washington)	(Wargacki et al., 2021)
Empty AAV8 viral particles	Mavis Agbandje-McKenna (University of Florida)	(Bennett et al., 2017; Wörner et al., 2021)
NativeMark Unstained Protein Standard	Thermo Fisher	LC0725

**Deposited data**		

Single-particle mass analysis data (MP)	This paper	figshare: https://doi.org/10.23644/uu.15132465
Single-particle mass analysis data (CDMS)	This paper	figshare: https://doi.org/10.23644/uu.15132465

**Software and algorithms**		

RawConverter (to MzXML) for CDMS data	(He et al., 2015)	http://fields.scripps.edu/rawconv/
Single-particle processing for CDMS data	(Wörner et al., 2020)	https://static-content.springer.com/esm/art%3A10.1038%2Fs41592-020-0770-7/MediaObjects/41592_2020_770_MOESM3_ESM.zip
DiscoverMP for processing of MP data	https://www.refeyn.com/refeyn-blog/categories/software	
Single-particle mass analysis (MP and CDMS)	This paper	figshare: https://doi.org/10.23644/uu.15132465

### Resource availability

#### Lead contact

Further information and other requests should be directed to and will be fulfilled by the lead contact, Albert J. R. Heck (A.J.R.Heck@uu.nl).

#### Materials availability

This study did not generate new unique reagents.

### Experimental model and subject details

Ribosome samples were gifted by the laboratory of Nenad Ban (ETH Zurich). Chloroplastic 70S were purified as previously described in detail by Bieri et al. (2017). Chloroplasts were extracted from fresh spinach leaves (*S. oleracea*) and lysed in buffer containing 10 mM Tris HCl pH 7.6, 25 mM KCl, 25 mM MgCl_2_, 2 mM DTT, 0.1 mM PMSF, 2 mM spermidine, 0.05 mM spermine, and 2% (w/v) Triton X-100. The lysate was centrifuged (25,350× g, 4°C, 30 min, Beckman Type 45Ti rotor). The supernatant was loaded onto 50% (w/v) sucrose cushion and centrifuged (101,390× g, 4°C, 15 h). The ribosome pellets were dissolved in monosome buffer containing 25 mM Tris HCl pH 7.6, 25 mM KCl, 25 mM MgOAc_2_, 2 mM DTT, 2 mM spermidine, 0.05 mM spermine. Then, they were layered onto 10–40% (w/v) sucrose gradient and centrifuged (51,610× g, 4°C, 15 h, Beckman Type SW-32Ti rotor). The fractions containing the chloroplastic 70S ribosomes were pooled and concentrated using Amicon Ultra-4 centrifugal filter (100 kDa molecular weight cutoff (MWCO)). Aliquots of the So70S sample were flash-frozen in liquid nitrogen and shipped by a dry shipper (Taylor-Wharton).

Human 80S ribosomes were purified as described previously by Quade et al. (2015). Frozen HEK-293 cells were thawed in lysis buffer (50 mM HEPES pH 7.6, 0.5% NP40, 6 mM MgCl_2_, 300 mM NaCl, 50 nM E-64, 20 μM leupeptin, 20 μM bestatin, 5 μM pepstatin A, 20 μM phenanthrolin, 1 μM PMSF and 2 mM DTT). The lysate was cleared by centrifugation (10 min, 12,000× g). The supernatant was loaded onto a 60% (w/v) sucrose cushion (50 mM HEPES pH 7.6, 50 mM KCl, 10 mM MgCl_2_, and 5 mM EDTA). Ribosomes were pelleted by centrifugation (50,000 r.p.m., 20 h, Ti70 rotor (Beckman Coulter)). The pellet was resuspended in resuspension buffer (50 mM HEPES pH 7.6, 6 mM MgCl2, 300 mM KCl, 1 mM DTT, 6.8% sucrose). The resuspended pellet was centrifuged again (20 min, 20,000× g), and the supernatant was treated with 1 mM puromycin for 30 min on ice. The ribosomal sample was then loaded onto a 10–40% (w/v) sucrose gradient in 50 mM HEPES pH 7.6, 2 mM MgCl_2_, 150 mM KCl and 1 mM DTT, and centrifuged (24,000 r.p.m., 17 h, SW32 rotor). The fractions containing 80S ribosomes were concentrated into 80S buffer (20 mM HEPES pH 7.6, 100 mM KCl, 5 mM MgCl_2_) using centrifuge filters (100,000 kDa MWCO (Sartorius)).

Human 40S and 60S ribosomal subunits were purified similarly to previously described protocols (Weisser et al., 2017). Frozen HEK293-6E cells were thawed and lysed in buffer containing 50 mM HEPES KOH pH 7.6, 300 mM NaCl, 6 mM MgAc_2_, 0.5% (w/v) NP-40, 5 μM E-64, 20 μM Leupeptin, 20 μM Bestatin, 5 μM Pepstatin A, 1 mM PMSF, and 2 mM DTT. The lysate was cleared by centrifugation for 20 min at 45,000× g and 4°C using a SS-34 rotor (Sorvall). The supernatant was loaded onto 60% (w/v) sucrose cushion and centrifuged for 20 h (257,000× g, 4°C) using a Beckman Type 70Ti rotor (Beckman-Coulter). The ribosome pellets were dissolved in resuspension buffer containing 50 mM HEPES KOH pH 7.6, 150 mM KCl, 6 mM MgAc_2_, and 2 mM DTT, layered onto 12-48% (w/v) sucrose gradient prepared with dissociation buffer (50 mM HEPES KOH pH 7.6, 500 mM KCl, 6 mM MgAc_2_, 2 mM DTT) and centrifuged for 18.5 h (78,000× g, 4°C, Beckman Type SW-32Ti rotor). The bands of the Hs60S and Hs40S ribosomal subunits were extracted with a syringe and concentrated using Amicon Ultra-15 centrifugal filters with 100 kDa MWCO (Merck Millipore). Aliquots of the Hs40S and Hs60S ribosome samples were flash-frozen in liquid nitrogen and shipped by a dry shipper (Taylor-Wharton).

The HCV IRES RNA were obtained similarly as previously described in detail by Quade et al. (2015). HCV IRES was produced by transcription *in vitro* using a linearized plasmid carrying the HCV IRES sequence. This was followed by denaturing polyacrylamide gel electrophoresis. The IRES was then buffer exchanged into water using centrifugal filters (10 kDa MWCO). Aliquots of HCV IRES RNA were flash-frozen in liquid nitrogen and shipped by a dry shipper (Taylor–Wharton).

I53-40 protein nanoparticles were obtained from the group of Neil P. King (University of Washington) and were prepared as recently described (Wargacki et al., 2021). Chemically competent *E.coli* strains were transformed using pET29b+ carrying designed I53-40 components. Cells were centrifuged and resuspended in the binding buffer (20 mM imidazole, 1 mM phenylmethanesulfonyl fluoride, 10 μM lysozyme, and 1 μM DNAse), followed by sonication. Cleared lysate was obtained by centrifugation and filtration of supernatant using a syringe filter (Millipore). Proteins of interest were purified via immobilized metal-affinity chromatography (IMAC) through 5 mL HisTrap Fast Flow columns (GE Healthcare). Fractions containing I53-40 were pooled and concentrated using centrifugal filters (10 kDa MWCO). Finally, assembled nanoparticles were purified via SEC on a Superose 6 10/300 gel filtration column.

Empty adeno-associated virus AAV8 particles were a gift from the laboratory of Mavis Agbandje-McKenna (University of Florida) and were produced as previously described (Bennett et al., 2017; Wörner et al., 2021). Briefly, fractions of samples were produced using a stable baculovirus/SF9 cell line, purified using an AVB sepharose column (GE Healthcare), and concentrated and loaded onto a 5–40% sucrose gradient for separation of empty AAV8 capsids. Centrifugation was performed at 151,000× g (3 h, 4°C, SW41 rotor). The samples were concentrated into 1XTD buffer in an Apollo concentrator (Orbital Biosciences). These I53-40 and AAV8 particles were used solely for high-mass calibration in mass photometry experiment.

### Method details

#### Sample preparation

Ribosomal particles were first buffer-exchanged into 1 M ammonium acetate solution (pH 7.5) with 2.5 mM magnesium acetate through a 30 kDa MW cutoff Bio-Rad P-30 Micro Bio-Spin columns, following vendor recommendations. Next, samples containing ribosomal particles were diluted and either directly used for mass spectrometry analysis at a final concentration of ∼ 100 nM or additionally further diluted to ∼ 2-5 nM for mass photometry analysis. For Hs40S ribosomal particles, final solution contained 250 mM ammonium acetate (pH 7.5) and 2.5 mM magnesium acetate. For Hs60S, So70S, and Hs80S ribosomal particles, final solution contained 750 mM ammonium acetate (pH 7.5) and 5 mM magnesium acetate.

Folded HCV IRES RNA samples were buffer-exchanged into 150 mM ammonium acetate solution (pH 7.5) using a 30 kDa Bio-Rad P-30 Micro Bio-Spin columns. Next, 100 nM Hs40S were mixed with different folds molar excess (∼ 2× and 4×) of HCV IRES RNA in ammonium acetate solution (pH 7.5) and incubated for 5 min at 37°C.

#### MP analysis of ribosomal particles

MP analysis was done using a Refeyn OneMP instrument (Oxford, UK). Contrast-to-mass calibration was achieved by measuring contrast of 4 proteins in the native marker protein standard mixture (NativeMark Unstained Protein Standard, Thermo Fisher), covering mass range from 0.1 up to 1.1 MDa. Four contrast values were used to generate a standard calibration curve, with following rounded average masses: 66, 146, 480, and 1048 kDa. The experiments were performed by using laboratory glass coverslips, which were thoroughly washed several times with Milli-Q water and isopropyl alcohol. A set of a few silicone gaskets placed onto the thoroughly dried glass surface was used for sample loading. Typically, an amount of 2 μL of ribosome-containing sample was mixed with 18 μL of 1 M ammonium acetate buffer with 2.5 mM Mg^2+^. Final concentration of the ribosomal particles was ∼ 2-5 nM. Movies of 6000 frames were recorded at a 100 Hz framerate using AcquireMP software and a large field-of-view acquisition settings. Single particles were automatically detected amounting to ∼ 3000 landing events per acquisition. All MP data was analyzed with DiscoverMP software to produce mass values for each detected particle and output as .csv file.

#### Orbitrap-based CDMS analysis of ribosomal particles

Samples were introduced into a Q Exactive mass spectrometer (QE-UHMR) through gold-coated borosilicate capillaries which were prepared in house. The following mass spectrometer settings were typically used. Capillary voltage: 1.3-1.6 V in positive ion mode. Collision gas: Xenon. In-source trapping voltage: −100 to −150 V. Noise level parameter: 0. The HCD voltage (100 V for Hs40S and Hs40S-IRES complexes, 150-200 V for Hs70S, 200-250 V for Hs60S and Hs80S) was optimized for maximal transmission and mass detection of intact ribosomes. A resolution of 280,000 at 200 *m/z* was set which resulted in a 1s transient time. The *m/z* calibration of instrument was performed using CsI clusters in the range between 350 and 12,000 *m/z*. After multi-scan acquisition (10 to 60 minutes), .RAW files were centroided and converted into the .mzXML format for data filtering and removal of dephasing ions, which occurred during the transient and caused splitting peaks (see also software and algorithms in the key resources table). These dephasing ions show intensity trails skewed toward lower values which can affect the charge state determination. Thus, all centroids which have an adjacent peak above the noise threshold within 5× peak FWHM in the *m/z* space were rejected. In this work, the noise threshold of intensity value was set at ∼ 8 elementary charges. After filtering, the remaining centroids of single-ion events are more confined in the intensity domain as well as their charge states. All measured intensities of centroids were normalized to 1 sec sample injection time. A calibration factor of 12.55 (normalized arbitrary intensities/charges) from the average of two replicates (12.52 and 12.59) in previous study (Wörner et al., 2020) was used to correlate the measured intensities and charges. After the calibration, the maximum deviation on charge determination does not exceed 1.6 charges in the range up to 250 charges. According to the determined charge state, a resulting formula *m* = *m/z* ∗ *z* - *z* was used to calculate the mass of each single ion in kDa.

#### Processing of mass distribution data from single-particle analysis

To facilitate comparison of the data obtained by single-particle methods we performed custom data processing starting with mass distributions as the input, which were either directly determined from contrast-to-mass standard calibration curve in the case of MP or converted from corresponding *m/z* and *z* values for CDMS. First, the raw data in mass window were processed with the kernel density estimation (KDE) function, from which local maxima and FWHMs were obtained for further processing. Next, mass distributions were mass binned with 50 and 5 kDa bins for CDMS and MP, respectively. The local maxima and FWHM were, then, used as input for Gaussian fitting in the binned data. Finally, mean mass of the fitted normal distribution and standard deviation were used to define detected ribosomes and co-purified assemblies. Mass values extracted through KDE function and Gaussian fitting are listed in the Table S5.

### Quantification and statistical analysis

Details regarding quantification and statistical analysis of single-particle mass data are provided in the method details section under “Processing of mass distribution data from single-particle analysis”. All relevant values for quantification and statistical analysis of mass distributions presented in this work (e.g. mean masses, standard deviation in Gaussian fitting, particle counts, etc.) are reported in Table S5.

## Data Availability

• The processed single-particle mass data have been deposited to figshare (figshare: https://doi.org/10.23644/uu.15132465) and are publicly available as of the date of publication. The corresponding folder for each figure or supplementary figure contains the displayed single-particle centroids from Orbitrap-based CDMS, as well as raw and processed single-particle data generated with mass photometry. • All original code has been deposited to figshare (figshare: https://doi.org/10.23644/uu.15132465) and is publicly available as of the date of publication. • Any additional information required to reanalyze the data reported in this paper is available from the lead contact upon request.
