# Supplementary material for: Single-particle mass analysis of intact ribosomes by mass photometry and Orbitrap-based charge detection mass spectrometry
Source: iScience. 2021 Oct 2;24(11):103211. doi: 10.1016/j.isci.2021.103211 (PMC8529500; doi:10.1016/j.isci.2021.103211)
Supplement: Document S1. Figures S1–S3 and Tables S1–S — 5 [file mmc1.pdf]

iScience, Volume *24*

Supplemental information

Single-particle mass analysis of intact ribosomes by mass photometry and Orbitrap-based charge detection mass spectrometry

Szu-Hsueh Lai, Sem Tamara, and Albert J.R. Heck

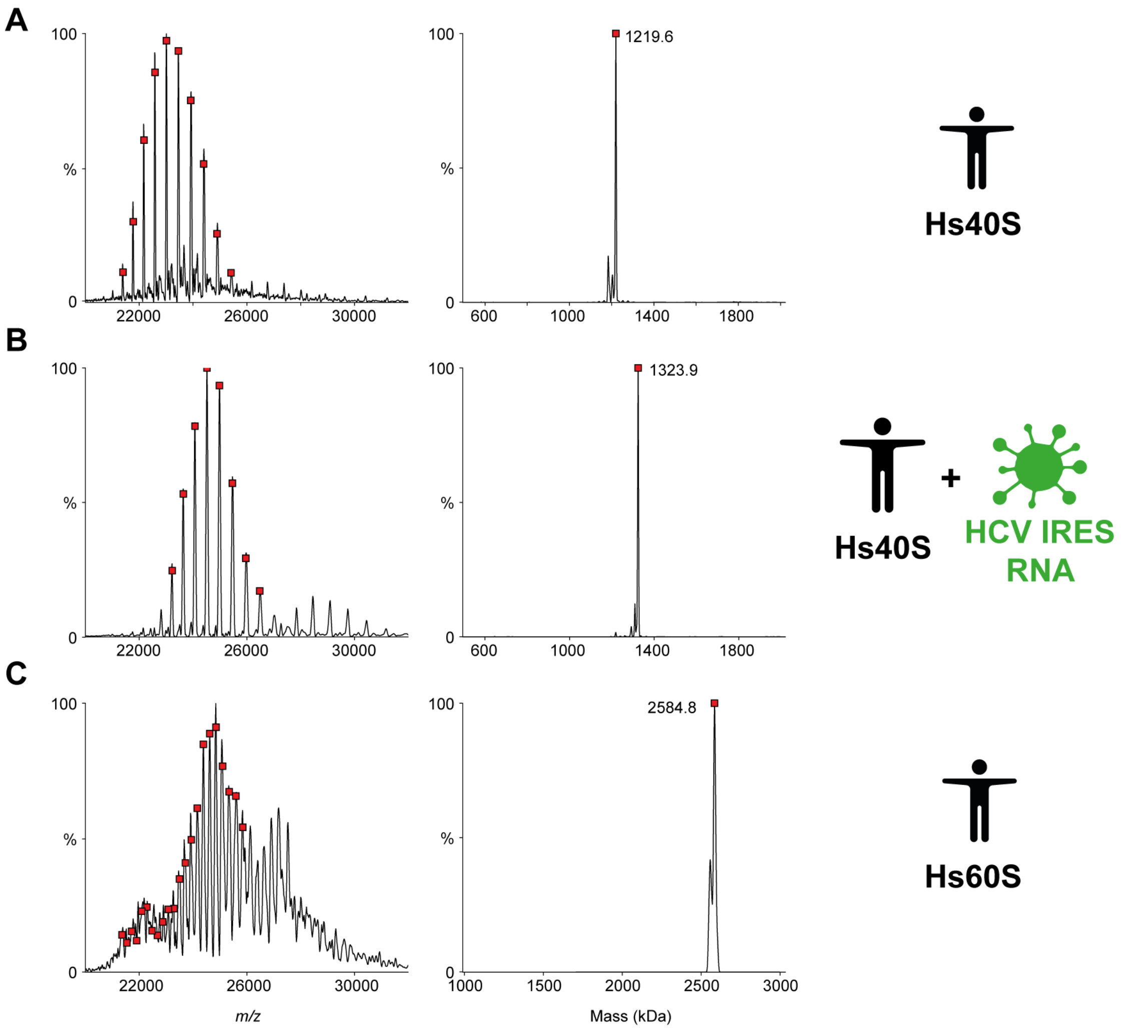

Figure S1. High-resolution native MS spectra of cytosolic ribosomal samples purified from human cells, related to Table 1.

Raw native mass spectra (left) and zero-charge deconvoluted mass spectra (right) of (A) Hs40S, (B) Hs40S in a complex with HCV IRES RNA, and (C) Hs60S ribosomal particles. Native mass spectra for (A) and (B) were acquired by spraying the samples from the solvent containing 25 mM triethylammonium acetate to facilitate stabilization and charge-reduction during high-energy desolvation (300 V HCD energy) on the Orbitrap UHMR mass spectrometer. Peaks corresponding to the major mass peak in each spectrum are annotated with red squares. Cartoons on the right are used to guide the eye of the reader.

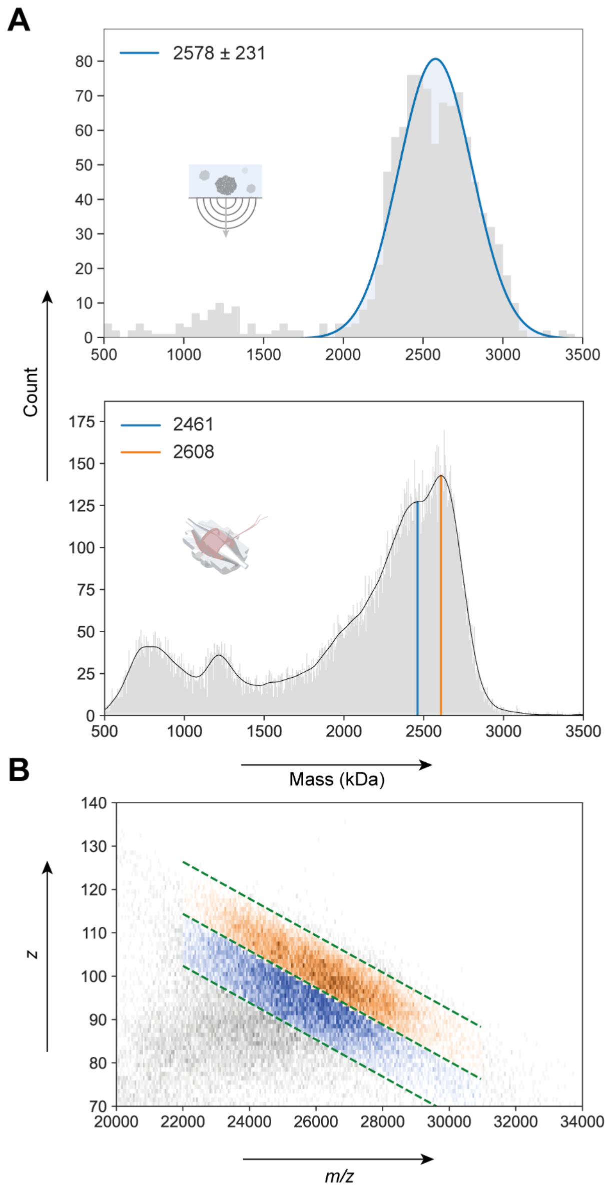

Figure S2. Single-particle analysis of Hs60S ribosomal particles by MP and CDMS, related to Figure 2.
(A) Mass distribution histograms obtained for the individually purified Hs60S sample using MP (top) and CDMS (bottom). The fitted mean mass and standard deviation of the most abundant species are provided for MP, while for CDMS the kernel density estimation is calculated and drawn over the mass distribution histogram with local maxima indicated in the mass range close to the theoretical mass of the Hs60S ribosomal subunit (2633 kDa).
(B) 2D-histogram representation of the Hs60S sample obtained by using CDMS. Color-coding is identical to the maxima annotation in (A)**.**

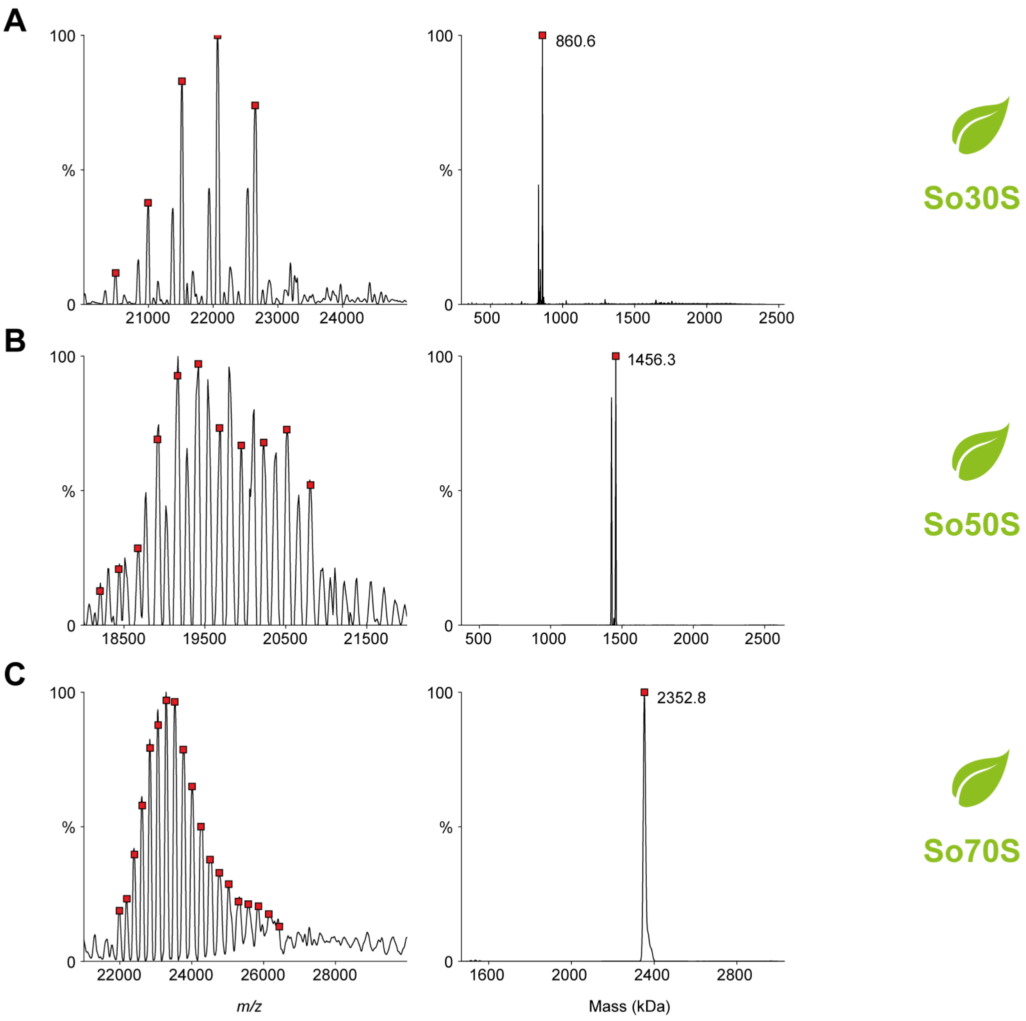

Figure S3. High-resolution native MS spectra of ribosomal samples purified from spinach chloroplasts, related to Table 1.
Raw native mass spectra (left) and zero-charge deconvoluted mass spectra (right) for (A) So30S, (B) So50S, and (C) So70S chloroplast ribosomal particles. Each spectrum was acquired by performing quadrupole isolation of the region of interest and the samples were sprayed from a solution either containing a (A,B) low concentration (0.5 mM) or (C) high concentration of Mg^{2+} (~5 mM). So30S ribosomal particles (A) were sprayed from a solvent containing 25 mM triethylammonium acetate to facilitate stabilization during high-energy desolvation (300 V HCD energy) on the Orbitrap UHMR mass spectrometer. Peaks corresponding to the major mass peak in each spectrum are annotated with red squares. Cartoons on the right are used to guide the eye of the reader.

Table S1. Theoretical and experimental masses of protein and rRNA subunits constituting the Hs40S ribosomal particles, related to Table 1.

Experimental masses were previously determined through top-down LC-MS/MS analysis as reported by Van de Waterbeemd et al. (Van De Waterbeemd et al., 2018)

Protein name	Uniprot Accession	Theoretical average mass (Da)	Experimental average mass (Da)
RS2	P15880	31193.23	31542.55
RS3	P23396	26557.12	26597.99
RS3A	P61247	29813.72	29812.36
RS4X	P62701	29466.53	29465.17
RS5	P46782	22876.4	22786.18
RS6	P62753	28680.64	28679.61
RS7	P62081	22126.85	22167.87
RS8	P62241	24073.99	24072.73
RS9	P46781	22460.24	22459.18
RS10	P46783	18897.77	18952.81
RS11	P62280	18299.53	18340.5
RS12	P25398	14383.74	14424.37
RS13	P62277	17091.11	17090.06
RS14	P62263	16141.51	16140.58
RS15	P62841	16908.91	16949.93
RS15A	P62244	14708.32	14707.24
RS16	P62249	16314.12	16313.1
RS17	P08708	15418.93	15418.1
RS18	P62269	17587.48	17628.57
RS19	P39019	15929.31	15928.37
RS20	P60866	13241.52	13282.51
RS21	P63220	9111.38	9152.45
RS23	P62266	15676.36	15691.28
RS24	P62847	15423.19	15465.56
RS25	P62851	13742.13	13638.96
RS26	P62854	12884.23	12883.21
RS27	P42677	9329.93	9370.82
RS27A	P62979	9418.09	9416.49
RS28	P62857	7841.03	7881.99
RS29	P62273	6545.57	6543.22
RS30	P62861	6647.86	6646.6
RSSA	P08865	32722.88	32763.64
RACK1	P63244	35076.73	34986.53
RL41	P62945	3456.35	3456.35
18S rRNA		603011.80	
**Σ**			**1213669**

Table S2. Theoretical and experimental masses of protein and rRNA subunits constituting Hs60S ribosomal particles, related to Table 1.

Experimental masses were previously determined through top-down LC-MS/MS analysis as reported by Van de Waterbeemd et al. (Van De Waterbeemd et al., 2018)

Protein name	Uniprot Accession	Theoretical average mass (Da)	Experimental average mass (Da)
RL10	P27635	24472.7	24444.25
RL10A	P62906	24700.12	24740.89
RL11	P62913	20121.19	20162.35
RL12	P30050	17818.59	17817.19
RL13	P26373	24261.48	24129.27
RL13A	P40429	23446.05	23486.94
RL14	P50914	23300.65	23655.58
RL15	P61313	24014.88	24013.7
RL17	P18621	21265.83	21264.79
RL18	Q07020	21503.3	21502.17
RL18A	Q02543	20762.36	20761.16
RL19	P84098	23465.97	23375.67
RL21	P46778	18433.64	18434.31
RL22	P35268	14655.8	14654.79
RL23	P62829	14865.44	14775.22
RL23A	P62750	17563.87	17604.92
RL24	P83731	17778.96	17778.24
RL26	P61254	17258.21	17256.96
RL27	P61353	15797.72	15707.54
RL27A	P46776	16430.19	16445.29
RL28	P46779	15616.31	15657.41
RL29	P47914	17620.88	17621.87
RL3	P39023	45977.7	45990.17
RL30	P62888	12784.05	12651.72
RL31	P62899	14462.86	14461.25
RL32	P62910	15728.61	15727.51
RL34	P49207	13161.76	13158.66
RL35	P42766	14420.28	14419.27
RL35A	P18077	12537.67	12447.52
RL36	Q9Y3U8	12122.51	12121.41
RL36A	P83881	12440.82	12320.88
RL37	P61927	10946.69	10943.54
RL37A	P61513	10144.06	10142.02
RL38	P63173	8086.7	8085.55
RL39	P62891	6275.49	6274.08

Protein name	Uniprot Accession	Theoretical average mass (Da)	Experimental average mass (Da)
RL4	P36578	47566.14	47606.94
RL40	P62987	6181.49	6221.76
RL41	P62945	3456.35	3456.16
RL5	P46777	34231.43	34229.8
RL6	Q02878	32596.73	32595.71
RL7	P18124	29225.77	29266.69
RL7A	P62424	29864.43	29863.07
RL8	P62917	27893.46	27908.29
RL9	P32969	21863.41	21862.33
RLA0	P05388	34273.51	34141.31
RLA1	P05386	11382.74	11423.76
RLA2	P05387	11664.94	11663.94
28S rRNA		1629202.4	
5S rRNA		38763	
5.8S rRNA		50654.1	
**Σ**			**2632893**

Table S3. Theoretical and experimental masses of protein and rRNA subunits constituting So30S ribosomal particles, related to Table 1.

Experimental masses were previously determined through top-down LC-MS/MS analysis as reported by Van de Waterbeemd et al. (Van De Waterbeemd et al., 2018)

Protein name	Theoretical average mass (Da)	Experimental average mass (Da)
pY	26805.2606	26804.9936
PSRP2	21665.1767	21664.9174
PSRP3	13794.1311	13794.2758
PSRP4	5173.83854	5173.31343
S01	40428.065	40428.2026
S02	26564.8581	26606.9096
S03	24796.9375	24796.4526
S04	23279.2362	23279.1049
S05	27630.4925	27689.2065
S06	14559.6936	14559.604
S07	17210.0237	17209.9409
S08	15368.0045	15367.7856
S09	17063.7765	17105.644
S10	15211.6267	15211.2817
S11	14766.2775	14766.7114
S12	13632.9913	13632.9243
S13	14536.9833	14536.7133
S14	11649.5426	11712.6928
S15	10753.6981	10752.8725
S16	10434.2099	10433.434
S17	12171.0565	12170.4614
S18	12053.0759	12052.5854
S19	10477.2435	10476.6704
S20	12918.9543	12918.7045
S21	12154.5883	12154.1176
16S rRNA	483683.2	
**Σ**		**908982.7**

Table S4. Theoretical and experimental masses of protein and rRNA subunits constituting So50S ribosomal particles, related to Table 1.
Experimental masses were previously determined through top-down LC-MS/MS analysis as reported by Van de Waterbeemd et al. (Van De Waterbeemd et al., 2018)

Protein name	Theoretical average mass (Da)	Experimental average mass (Da)
L01	30443.1855	30443.1855
L02	29661.2464	29661.2464
L03	24074.2279	24074.2279
L04	27163.7	27225.7281
L05	24210.1445	24210.1445
L06	20226.5493	20226.5493
L09	17635.9005	17635.9005
L10	20310.5897	20310.5897
L11	16667.8094	16667.8094
L12	13814.7232	13814.7232
L13	23361.9846	23361.9846
L14	13464.7332	13464.7332
L15	22329.6623	22329.662
L16	15297.9925	15297.9925
L17	13434.8045	13434.8045
L18	13701.7866	13701.7866
L19	17566.343	17566.343
L20	15557.3818	15557.3818
L21	22562.8355	22763.0298
L22	23114.3406	23245.5332
L23	13553.8013	13553.801
L24	16408.0505	16408.0505
L27	14058.222	14058.222
L28	8992.59724	8992.59724
L29	12972.172	12972.172
L31	10781.4944	10781.4944
L32	6503.73784	6503.73784
L33	7516.84534	7516.84534
L34	6768.04684	6768.04684
L35	8433.06514	8433.06514
L36	4401.39584	4401.39584
PSRP5	9255.03074	9255.03074
PSRP6	7387.27114	7387.27114
RRF	21837.9905	21837.9905
23S rRNA	911551	
5S rRNA	39219.2	
4S rRNA	34539.2	
**Σ**		**1539172**

Table S5. Masses and number of particles determined for ribosomal particles using the single-particle approaches, Orbitrap-based CDMS and MP, related to Figures 2, 3, and 4.
Counts normalized column represents fractions of total particle counts that were quantified for specific peaks.

Method	Sample	Particle name	Theoretical mass (kDa)	Mass from KDE (kDa)	FWHM (kDa)	Mean mass of the Gaussian fit (kDa)	Standard deviation of the Gaussian fit (kDa)	Counts normalized (%)	Counts (peak)	Counts (total)	Collection time (min)
CDMS	Hs40S	Hs40S	1214	1217	140	1215	48	47	3737	7951	17
	Hs80S	Hs40S	1214	1215	358	1210	117	19	6477	34123	31
		Hs60S	2633	2361	362	2367	149	56	19010		
		Hs80S	3847	3890	420	3867	163	11	3751		
	So70S	So30S	909	802	198	805	72	32	30092	92703	41
		So50S	1539	1239	348	1238	128	37	34187		
		So70S	2448	2380	228	2381	88	20	18730		
MP	Hs40S	Hs40S	1214	1226	312	1211	110	82	1000	1218	5
	Hs80S	Hs40S	1214	1217	480	1215	109	25	141	573	5
		Hs60S	2633	2485	622	2491	224	50	284		
		Hs80S	3847	3805	1017	3830	311	19	111		
	So70S	So30S	909	850	339	848	111	25	213	844	5
		So50S	1539	1475	473	1483	158	25	212		
		So70S	2448	2403	640	2344	209	45	377		
